# Comparative electroencephalography analysis: Marathon runners during tapering versus sedentary controls reveals no significant differences

**DOI:** 10.1002/brb3.3480

**Published:** 2024-04-28

**Authors:** J. Moussiopoulou, M. Handrack‐Bonnet, B. Pross, O. Pogarell, D. Keeser, M. Halle, P. Falkai, J. Scherr, A. Hasan, A. Roeh

**Affiliations:** ^1^ Department of Psychiatry and Psychotherapy LMU University Hospital, LMU Munich Munich Germany; ^2^ Medical Faculty, Department of Psychiatry, Psychotherapy and Psychosomatics, Bezirkskrankenhaus Augsburg University of Augsburg Augsburg Germany; ^3^ Department of Prevention and Sports Medicine Klinikum rechts der Isar, Technische Universitaet Muenchen Munich Germany; ^4^ Partner Site Munich Heart Alliance Deutsches Zentrum für Herz‐ und Kreislauf‐Forschung (DZHK) e.V. (German Center for Cardiovascular Research) Munich Germany; ^5^ University Center for Preventive and Sports Medicine, Balgrist University Hospital, University of Zurich Zurich Switzerland

**Keywords:** EEG, marathon, physical activity, plasticity, running

## Abstract

**Introduction:**

Previous studies described various adaptive neuroplastic brain changes associated with physical activity (PA). EEG studies focused mostly on effects during or shortly after short bouts of exercise. This is the first study to investigate the capability of EEG to display PA‐induced long‐lasting plasticity in runners compared to a sedentary control group.

**Methods:**

Thirty trained runners and 30 age‐ and sex‐matched sedentary controls (SC) were included as a subpopulation of the ReCaP (Running effects on Cognition and Plasticity) study. PA was measured with the International Physical Activity Questionnaire (IPAQ). Resting‐state EEG of the runners was recorded in the tapering phase of the training for the Munich marathon 2017. Power spectrum analyses were conducted using standardized low‐resolution electromagnetic tomography (sLORETA) and included the following frequency bands: delta: 1.5–6 Hz, theta: 6.5–8.0 Hz, alpha1: 8.5–10 Hz, alpha2: 10.5–12.0 Hz, beta1: 12.5–18.0 Hz, beta2: 18.5–21.0 Hz, beta3: 21.5–30.0 Hz, and total power (1.5–30 Hz).

**Results:**

PA (IPAQ) and BMI differed significantly between the groups. The other included demographic parameters were comparable. Statistical nonparametric mapping showed no significant power differences in EEG between the groups.

**Discussion:**

Heterogeneity in study protocols, especially in time intervals between exercise cessation and EEG recordings and juxtaposition of acute exercise‐induced effects on EEG in previous studies, could be possible reasons for the differences in results. Future studies should record EEG at different time points after exercise cessation and in a broader spectrum of exercise intensities and forms to further explore the capability of EEG in displaying long‐term exercise‐induced plasticity.

## Graphical abstract

No long‐term EEG differences were detected in marathon runners versus sedentary controls in this naturalistic study. Previous studies, reporting EEG differences, mostly investigated acute postexercise effects (interventional designs). Methodic heterogeneity hinders comparability in previous studies; this study addresses this scientific problem and paves the way for methodic preciseness when investigating EEG effects of PA.

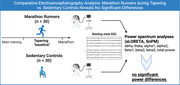

## 1 INTRODUCTION

Physical activity (PA) and exercise have been extensively studied for their effects on physical health, particularly on the cardiovascular system (Varghese et al., 2016). Additionally, neurophysiological adaptations resulting from both chronic and acute exercise have been well documented. Chronic exercise induces neuroplasticity, involving molecular, cellular, structural, and functional changes (El‐Sayes et al., 2019). Notable effects include elevated levels of brain‐derived neurotrophic factor (BDNF), insulin‐like growth factor 1 (IGF‐1), vascular endothelial growth factor (VEGF), and receptors, promoting processes such as gliogenesis, neurogenesis (Pereira et al., 2007), synaptogenesis, and angiogenesis. These adaptations contribute to increases in gray matter volume (GMV) particularly in regions relevant for cognitive functions like the hippocampus (Firth et al., 2018) and white matter volume (WMV), along with enhanced neural and receptor activity, leading to improvements in cognitive (De Sousa Fernandes et al., 2020) and motor functions (El‐Sayes et al., 2019). Moreover, higher levels of PA have been associated with greater structural and functional connectivity (FC) as observed through neuroimaging techniques (Ruotsalainen et al., 2021; Soldan et al., 2022; Stillman et al., 2018), in networks critical for maintaining multiple aspects of overall brain health and hindering cognitive decline (Stillman et al., 2018). Despite these findings, the precise mechanisms underpinning exercise‐induced neuroplasticity remain incompletely understood (El‐Sayes et al., 2019).

Despite the wealth of knowledge derived from these (neuroimaging) techniques, there is a need to explore simpler and more accessible methods. Electroencephalography (EEG), offering portability, quickness, and cost‐effectiveness, presents itself as a valuable tool for investigating brain electrocortical activity and cortical reorganization with high temporal resolution. Identifying EEG correlates of PA effects could enhance our understanding and establish EEG as a feasible proxy for detecting neurophysiological changes, especially when more complex methods like magnetic resonance imaging (MRI) are less feasible.

To ensure a clear understanding and to enable comparability between studies, it is crucial to define terms commonly used in the literature. “PA” encompasses any bodily movement resulting in energy expenditure, while “exercise” is a planned, structured and repetitive subset of PA aimed at improving or maintaining physical fitness (Caspersen et al., 1985). “Acute exercise” refers to a single bout or session lasting from a few minutes to a few hours (Jee, 2020), triggering molecular and functional neuroplasticity, associated with heightened oxygen and glucose metabolism, elevated neurotransmitter concentration, and increased cerebral blood flow (CBF) (El‐Sayes et al., 2019). This is in contrast to “chronic exercise,” involving long‐term, regular activity (Jee, 2020). Despite the focus on acute effects in current research, observed during or shortly after exercise cessation in pre‐post interventional study designs (Boutcher & Landers, 1988; Crabbe & Dishman, 2004; Schneider et al., 2009; Schneider et al., 2009; Woo et al., 2009), the longevity and subacute/chronic effects of exercise‐induced neuroplasticity, measured hours or days after exercise cessation, further including the cellular and structural aspects and lacking the acute metabolic elements (El‐Sayes et al., 2019), remain understudied. Additionally, the intensity of exercise, categorized as low, moderate, or vigorous, plays a crucial role (Ikuta et al., 2019). While moderate exercise has established benefits, the neurophysiological adaptations following prolonged and vigorous exercise are less clear.

Some of the studies investigating effects of acute exercise found an acute increase in alpha power during or immediately post exercise (Boutcher & Landers, 1988; Brümmer et al., 2011; Crabbe & Dishman, 2004; Honzák et al., 1985; Schneider et al., 2009). Alpha activity, the dominant resting‐state frequency typically ranging from 8 to 13 Hz (Newson & Thiagarajan, 2018), has been correlated with enhanced cognitive performance (Richard Clark et al., 2004). It has also been reported to be declined in neuropsychiatric disorders associated with cognitive deficits (Newson & Thiagarajan, 2018; Ramsay et al., 2021) and has even been proposed as a biomarker of early cognitive decline (Lejko et al., 2020). Despite these observations, the reported acute effects exhibited overall heterogeneity in both frequency bands and brain localization. This heterogeneity could be confounded by the methodological differences in three main variables: type, duration/intensity of exercise and the time lapse between exercise cessation and EEG recordings. All of the aforementioned studies explored the immediate (acute) effect of PA (mostly single acute bouts of exercise) on brain waves. Investigations of subacute and long‐term adaptations induced by chronic aerobic exercise in EEG, reflecting the long‐lasting changes in neuroplasticity, are lacking.

Addressing these gaps in knowledge, this study compares the EEG of marathon‐training runners to a sedentary control (SC) group. To assess the long‐term effects of chronic exercise, as opposed to the acute effects, we conducted recordings with a significant time lapse after the last training session during the tapering phase, which occurs in the weeks before a race. The tapering phase involves a deliberate, strategic reduction in training volume and intensity, aimed at facilitating recovery and enhancing performance (Haugen et al., 2022). In this study, it serves as a “baseline” since the intensive training period could be interpreted as an intervention. This is the first study to investigate the effects of prolonged and intense aerobic exercise on EEG, not immediately after an exercise intervention, as done in previous studies, but after a relevant time span from exercise cessation. Thus, long‐lasting electrocortical adaptations are displayed in a naturalistic design. Building on reported acute effects and given the established evidence of neurophysiological adaptations to chronic and acute exercise along with the recognized association of enhanced alpha power and improvements in connectivity and cognition, we hypothesize that prolonged and intense aerobic exercise, characteristic of marathon training, may manifest in higher alpha power in runners compared to SC measured by EEG. By including the other frequency bands (1.5–30 Hz) in an exploratory approach we strive for an unbiased assessment. Our study diverges by examining EEG after a relevant time span from exercise cessation, contributing to a nuanced understanding of enduring exercise‐induced neuroplasticity beyond immediate postexercise assessments.

## 2 MATERIALS AND METHODS

### 2.1 Subjects

This study was a subanalysis and the cohort was a subcohort of the ReCaP trial (Running effects on Cognition and Plasticity), a longitudinal observational study of marathon runners (Roeh et al., 2020). For the EEG analyses, 30 runners with successful registration for the Munich marathon 2017 (08.10.2017) and who had experience in endurance training (at least one finished half marathon) were recruited by announcements in local newspapers, local running groups, and newsletters of the local organizer of the Munich marathon. Exclusion criteria were severe internal, neurological, and psychiatric illnesses, BMI ≥30 kg/m^2^, regular drug‐abuse and insufficient knowledge of German language.

Regarding the age‐ and sex‐matched sedentary control group (SC, *N* = 30), recruited via announcements in local newspapers and other channels (e.g., social media), prerequisites were as little physical activity as possible (less than 25 min of self‐reported PA a day as definition of a sedentary lifestyle (De León et al., 2007) including everyday activities such as cycling to work) and no experience in endurance running. The other inclusion criteria (age, knowledge of German, no severe illnesses, BMI < 30 kg/m^2^) were identical to those of the runners. Prior to inclusion in the study, all participants provided written informed consent. The study protocol was approved by the ethics committees of both the Ludwig‐Maximilian University Munich (approval reference number 17–148) and the Technical University Munich (approval reference number 218/17).

### 2.2 Demographic data, PA assessments

Apart from the acquisition of basic demographic data (age, weight, BMI, smoking history, sex, education), the full long version of the International Physical Activity Questionnaire (IPAQ) (Booth, 2000; Craig et al., 2003) was conducted in both groups, presenting detailed information about daily physical exercise as well as PA in daily routines. As further indicators for fitness, maximal oxygen consumption (VO2max) (Bacon et al., 2013), measured through spiroergometry, self‐reported yearly and weekly running volume (assessed during MA training) and automatically recorded marathon performance (Munich marathon, 08.10.2017) were assessed in the runner group.

### 2.3 EEG measurements and processing

To avoid overlapping acute changes in EEG caused by the marathon in postmarathon surveys, we compared EEG recordings of runners, measured during the tapering phase, 14 to 4 days before the Munich marathon (08.10.2017), with EEG recordings of SC, conducted between July 2017 and January 2018. Runners and SC were instructed not to perform any training on the day of the EEG recordings.

EEG‐actiCaps (BrainProducts GmbH) with 32 electrodes adapted to the head circumference of the participant were used, arranged in accordance with the international 10–20 system (Jasper, 1958) at FP1, FP2, F7, F3, Fz, F4, F8, FC5, FC1, FC2, FC6, T7, C3, Cz, C4, T8, TP9, CP5, CP1, CP2, CP6, TP10, P7, P3, Pz, P4, P8, PO9, O1, Oz, O2, and PO10. Resting‐state EEG activity was recorded for six to 7 min. The impedance of the skin electrode was kept below 5 kΩ. All recordings were performed with the subject in a resting, mentally relaxed supine position with eyes closed in a sound‐ and light‐attenuated room in our laboratory at the University Hospital LMU Munich. Alpha‐blocking (Berger effect) was performed (instructing subjects to close and open their eyes) before and at the beginning of EEG recording to ensure the quality of the recorded data. During EEG recordings, participants were supervised by an investigator. After recording, EEG data were imported to BrainVision Analyzer Version 2.0 (Brain Products GmbH, Gilching) to be processed via different filters and applications (70 Hz low‐pass filter; 1 Hz high‐pass filter; 50 Hz notch‐filter, and a 250 Hz sampling rate) and to manually remove artifacts (that reflect significant nonbrain activity that contaminate EEG data, such as electrode misplacement, cable movements, muscle contractions (Jiang et al., 2019)) in the “Raw Data Inspection” processing mode, under supervision of an expert in the field. From each recording, the first 140 artifact‐free EEG segments (one segment = 2000 ms) were used for further analysis and exported to the text ASCII format.

### 2.4 Data analyses

Current source density analysis was performed in 3‐D Talairach/MNI space (Talairach & Tournoux, 1988) using standardized low‐resolution electromagnetic tomography (sLORETA) (Pascual‐Marqui, 2002). The version used in our study is an advanced version of LORETA (Pascual‐Marqui et al., 2002) and estimates the current source density distribution and source localization in 6.239 cortical gray matter voxels, with a cubic voxel size of 5 mm^3^. The sLORETA statistical nonparametric mapping tool (SnPM) was used, based on a paired voxel‐by‐voxel log‐*F*‐ratio test using 5000 randomizations, for comparison (Villafaina et al., 2019). It is based on estimating the empirical probability distribution for the max‐statistic under the null hypothesis by randomization. The analysis included the following frequency bands: delta (1.5–6 Hz), theta (6.5–8.0 Hz), alpha1 (8.5–10 Hz), alpha2 (10.5–12.0 Hz), beta1 (12.5–18.0 Hz), beta2 (18.5–21.0 Hz), beta3 (21.5–30.0 Hz), and total power (1.5–30 Hz). Statistical significance levels were set to *p* = .05 and *p* = .10 (significant trend). The null hypothesis of no activation anywhere in the brain was rejected if at least one test value was above the critical threshold for *p* = .05 (Eugene et al., 2015; Horacek et al., 2007). Correction for multiple testing was calculated with the nonparametric randomization methodology (Nichols & Holmes, 2002) already implemented in the sLORETA software package (Pascual‐Marqui, 2002). This methodology corrects for multiple testing (i.e., for the collection of tests performed for all electrodes and voxels and for all time samples). Due to the nonparametric nature of the method, its validity need not rely on any assumption of Gaussian distribution (Pascual‐Marqui, 2002). To compare demographic data and IPAQ results between runners and sedentary controls, we used independent t tests and χ^2^ tests in SPSS 26 (IBM SPSS Statistics, Version 26) with a significance level of *p* = .05. Mean values and standard deviation (SD) were calculated using descriptive statistics. Conditions for statistical tests such as homogeneity of variances were given.

## 3 RESULTS

Thirty (4 female; 45 ± 9 years) participants of the Munich Marathon 2017 underwent 6‐min resting‐state EEG measures. The SC group consisted of thirty (5 female; 40 ± 12 years) healthy subjects. Runners had finished at least one half marathon prior to the study (inclusion criterion), ran a mean of 51.15 km per week (SD = 21.67, *N* = 26, min = 13 km, max = 90 km) during Marathon training, and a mean of 1933 km per year (SD = 457.19, *N* = 19, min = 400 km, max = 3600 km) had a mean VO2max of 47.95 mL/kg/min (SD = 4.47, *N* = 29, min = 38.3 mL/kg/min, max = 58.8 mL/kg/min) and mean Marathon performance was 220.80 min (SD = 29.45, *N* = 25, min = 175.10, max = 283.48). As expected, IPAQ and BMI differed significantly between groups. Table 1 displays demographic data and PA.

**TABLE 1:** Baseline characteristics of the marathon group and sedentary control group.

	*N* (MR/SC)	Runners		SC		Runners vs. SC		
						χ^2^	*df*	*p*
Demographics								
Sex (m: f)	60 (30/30)	26:4		25:5		0.13	1	.718
Smoking (yes: no)	56 (28/28)	1:27		5:23		2.99	1	.084
		Mean	*SD*	Mean	*SD*	*t* value	*df*	*p*
Age (years)	60 (30/30)	44.57	9.48	39.83	11.60	1.73	58	.089
Education (years)	56 (27/29)	15.52	4.01	14.50	3.47	1.02	54	.313
BMI (kg/m^2^)	58 (29/29)	23.15	2.29	25.08	2.54	–3.04	56	.004*
IPAQ	56 (29/27)	6290.67	5030.39	2495.69	4428.28	2.99	54	.004*

*Note*: *N* < 60 means missing data.

MR = marathon runners, SC = sedentary control group, BMI = body mass index, IPAQ = International Physical Activity Questionnaire‐Long Version (in MET‐minutes per week (Metabolic Equivalent)).

* = statistical significance.

SLORETA's nonparametric mapping (SnPM) was applied to determine and localize changes in frequency bands. Table 2 shows the test results of each compared frequency band. Overall, no statistically significant differences were observed in power spectra analyses of control subjects and runners.

**TABLE 2:** Log‐*F*‐ratio marathon group versus sedentary control group, sLORETA.

Frequency bands	Log‐*F*‐ratio	*p*
Delta	0.6459	>.1
Theta	0.6915	>.1
Alpha 1	0.9125	>.1
Alpha 2	–0.9632	>.1
Beta 1	0.6234	>.1
Beta 2	0.5420	>.1
Beta 3	0.8035	>.1
Total power	0.6294	>.1

## 4 DISCUSSION

The present study marks the first attempt to compare EEG resting‐state frequency bands in endurance runners undergoing intensive regular training with a sedentary control group, investigating enduring electrocortical adaptations to physical activity (PA) in a naturalistic design. Despite the athletic condition of our runners, our findings did not reveal detectable physiological EEG changes when compared to the sedentary control group.

We selected EEG as a valuable and practical tool for prognostic and diagnostic purposes, given its reflection of cortical plasticity (Manganotti et al., 2022). Changes in power bands across different cortical areas occur in response to various processes such as fatigue and reactions to training stimuli, influencing learning processes (Manganotti et al., 2022). Nonetheless, changes in EEG resulting from exercise interventions are elusive and challenging to replicate (Gramkow et al., 2020). Previous studies have reported heterogeneous results regarding the immediate effects of acute exercise on EEG measures, with a frequent observation of increased alpha activity (Crabbe & Dishman, 2004), particularly observed during and up to approximately 6 min after exercise cessation (Crabbe & Dishman, 2004), suggesting an acute (rather than long‐lasting) postexercise effect.

Limited studies have explored the effects of prolonged exercise on EEG recordings. For instance, Honzák et al. (1985) observed a decrease in theta activity and an increase in slow alpha component and subtheta activity in marathon runners after a 2‐week endurance training period, attributing this to fatigue and poor oxygen and glucose supply to the brain. Another review on plasticity‐inducing interventions, including yoga, demonstrated an increase in alpha activity in longitudinal interventional groups, linked to improvements in cognition, memory, mood, and anxiety (Desai et al., 2015). The overall yoga‐induced effects were, however, also heterogeneous, and most included studies were interventional, with a small number of subjects and only a few included a control group. While these studies examined longitudinal effects, they primarily did so within an interventional period (PA/ yoga), frequently involving regular pre‐/postsession measurements throughout this period. However, there was a lack of reporting, or consideration for, a relevant hiatus between the cessation of PA or yoga and EEG recordings. This absence of a relevant break makes it challenging to differentiate between acute and long‐lasting effects.

To explore the enduring effects of PA on electrocortical activity, reflecting long‐term neuroplasticity without the interference of acute effects, a significant time span between exercise termination and EEG recordings was crucial in our study design. Similar considerations apply to other modalities assessing neuroplasticity, such as transcranial magnetic stimulation (TMS) (Jannati et al., 2023). While TMS‐induced neuroplasticity, measured through motor evoked potentials (MEPs), is detectable for a limited time poststimulation (Jannati et al., 2023), its persistence can vary, echoing our approach to evaluate enduring exercise‐induced EEG effects.

Despite established evidence indicating long‐term neuroplastic adaptations to PA, including increased gray matter (e.g., in the hippocampus), enhanced synaptic plasticity, connectivity, and spatial memory function (De Sousa Fernandes et al., 2020; El‐Sayes et al., 2019), and despite the known associations between enhanced cognition, connectivity, and elevated alpha activity, our study did not identify a correlate of long‐term plasticity in the form of elevated alpha power.

While exercise‐induced changes in EEG have been previously documented to be of short duration (Bailey et al., 2008; Crabbe & Dishman, 2004), it has also been reported that the effects are more pronounced at higher levels of PA intensity (Schneider et al., 2009). Similar observations have been reported in functional connectivity (Ikuta et al., 2019). These observations align with our hypothesis of identifying enduring alterations in EEG frequencies among individuals with high levels of physical activity.

Our findings, showing no differences between runners and the sedentary control group, likely result from the chosen timepoint (tapering), with a significant time lapse between the last exercise bout and EEG recordings. This underscores the transient nature of previously reported acute EEG effects of exercise, mainly driven by acute rather than chronic exercise, due to the different physiological underpinnings of the two (El‐Sayes et al., 2019).

The physiological explanations for temporary acute exercise‐induced EEG effects are complex and multifaceted. One possible explanation is the temporary increase in central nervous activation, due to increased somatosensory afferents during and shortly after exercise (Krause et al., 1983). Once subsided, the temporarily increased cortical activation likely returns to baseline. Another possible mechanistic explanation are emotional responses to exercise. One study reported that exercise mode, intensity, and individual preferences influence EEG effects (Brümmer et al., 2011). As these activation patterns are directly connected to the ongoing or recently completed acute exercise, they diminish when the emotional response wanes. Fatigue induced by exercise, particularly central fatigue, can contribute to the temporary nature of EEG effects (Brümmer et al., 2011; Dalsgaard & Secher, 2007). Central fatigue pertains to situations in which the capacity of the central nervous system to activate motoneurons restricts the expression of strength (Dalsgaard & Secher, 2007) and its duration after exercise can vary depending on several factors but usually lasts between minutes and hours postexercise (Carroll et al., 2017). Although central fatigue correlates with EEG changes (e.g., increased alpha power) (Ghorbani & Clark, 2021) the present study's recordings were not during a fatigue stage, possibly explaining the negative results. Exercise‐induced changes in mood (Roeh et al., 2020; Schoenfeld & Swanson, 2021), linked to increased cortical excitation, are another significant factor. Despite heterogeneous evidence on EEG effects resulting from exercise‐induced mood changes (Lattari et al., 2014) some reported acute effects may be associated with mood changes that subside hours after exercise (Peluso & Guerra de Andrade, 2005). Additional mechanisms contributing to temporary EEG effects encompass alterations in cerebral blood flow (Secher et al., 2008; Smith & Ainslie, 2017), overall metabolism, and neurotransmitters (e.g., catecholamines (Stock et al., 1996), endorphins (Schoenfeld & Swanson, 2021)). All these processes are probably not separate mechanisms, but complexly and dynamically intertwined with each other and seem to be reflected by the acute EEG effects reported in the literature. After hours or days after exercise cessation, they subside, possibly explaining the negative findings in the here presented study.

To prevent misinterpretation, the results of the present study (lack of group differences) should be interpreted strictly within the methodological context, considering the timepoint of EEG recording (hours/days after exercise cessation during tapering), or the underrepresentation of biological females (as they show a greater propensity for neuroplasticity (El‐Sayes et al., 2019)) and should be evaluated within the context of the limitations.

Several limitations should be considered. The low spatial resolution of EEG may underrepresent potential changes. The absence of EEG recordings during or immediately after acute training limits our understanding of exercise's acute effects. While changes in EEG might have been detectable shortly after PA, our study prioritized a naturalistic design reflecting runners’ everyday reality, focusing on long‐term effects rather than acute interventions. A combination of EEG during or shortly after training and in the tapering phase would enhance interpretative robustness. The SC group lacked VO2max assessments, hindering objective fitness evaluation alongside the self‐reported IPAQ data, which covers only the past 7 days, potentially missing more active periods. Further studies should conduct objective fitness assessments to ensure a significant differentiation of the cohorts. The runners group's heterogeneity in PA habits (showing wide dispersion of IPAQ values) and the absence of a standardized training protocol reduce intersubject comparability. A more precise assessment of the exact training routine would facilitate the interpretation of the results. Variability in tapering strategy and the time span between intensive training and EEG recordings among runners and slight circadian inconsistencies in recording times may impact results. The condition of closed eyes, chosen for enhanced alpha activity, limits comparability with studies using open eyes. Although our sample size (*N* = 60) is larger than some studies, larger cohorts are desirable for more robust results in the future.

## 5 CONCLUSION

Although persisting exercise‐induced central adaptations were registered in other modalities (e.g., MRI) and in EEG shortly after acute bouts of PA, we could not detect EEG differences between runners and sedentary controls in our study. Future studies should further explore the capability of EEG in displaying exercise‐induced plasticity. To do so, further improvement of methodological preciseness is needed, that is, recording EEG at different time points during and after PA cessation to better understand the differences between acute and possible long‐term EEG adaptations. Additionally, combining EEG with other modalities, such as neuroimaging, would allow a better understanding of the underlying mechanisms.

## AUTHOR CONTRIBUTIONS

AH, AR, JS, PF, and MaH conceptualized and supervised the study (conceptualization, project administration). AR and AH supervised the study (supervision). BP, MH, and JM collected the EEG data (investigation). JM and BP curated the data (data curation). JM and BP performed the statistical analyses (methodology, software, formal analysis). OP and DK provided support in all aspects of data collection and analysis (formal analysis, supervision). MH and JM wrote the manuscript (writing—original draft). JM conducted the main revision. All authors revised the manuscript and approved the final version (Writing—review and editing). All authors provided critical feedback and helped shape the research, analysis, and manuscript. All authors agreed to be accountable for all aspects of the work in ensuring that questions related to the accuracy or integrity of any part of the work are appropriately investigated and resolved.

## CONFLICT OF INTEREST STATEMENT

The authors of this manuscript have no conflicts of interest to report.

### PEER REVIEW

The peer review history for this article is available at https://publons.com/publon/10.1002/brb3.3480.

## Data Availability

The data that support the findings of this study are available upon request from the corresponding author. Moreover, we offer to publish the raw EEG data in BIDS format after acceptance of our article at a platform such as OSF.
